# Additive positive effect of warming and elevated nitrogen deposition on *Sphagnum* biomass production at mid-latitudes

**DOI:** 10.1038/s41598-024-67614-5

**Published:** 2024-07-22

**Authors:** Yoshitaka Oishi

**Affiliations:** Center for Arts and Sciences, Fukui Prefectural University, 4-1-1 Kenjojima, Matsuoka, Yoshida-gun, Eiheiji-cho, Fukui 910-1195 Japan

**Keywords:** *Sphagnum* moss, Global warming, Nitrogen deposition, Carbon isotope ratio, Chlorophyll fluorescence, Mid-latitude, Climate-change ecology, Wetlands ecology

## Abstract

Global warming and increased atmospheric nitrogen (N) deposition can adversely impact *Sphagnum* moss populations and ecological functions in peatlands. Based on the anticipated increases in temperature and N levels at global scale, we investigated the effects of simultaneous warming and N treatment on growth and ecophysiological activity of *Sphagnum papillosum*, a predominant moss at mid-latitudes, utilizing a growth chamber experiment. Warming treatments increased the maximum yield of photosystem II (Fv/Fm) of *S. papillosum* while decreasing the stable carbon isotope ratio. However, warming treatment alone did not cause significant changes in the biomass increase from that of the control. Regarding N treatment, the low N treatment decreased Fv/Fm under the current temperature but did not affect the biomass increase. In contrast to these results, a simultaneous warming and high N treatment significantly enhanced the biomass production compared to that of the control, exhibiting additive effect of warming and high N treatment on *Sphagnum* biomass production. These responses were attributed to the improved photosynthetic performances by warming and N treatment. The results of this study contribute to the prediction of *Sphagnum* responses to warming and changes in N deposition.

## Introduction

Peatlands play important ecosystem roles such as global carbon storage. Approximately one-third of the world’s soil carbon is accumulated in peatlands^1^. The belowground carbon storage in peatlands is largely dependent on peat-forming mosses, particularly *Sphagnum* mosses^2^, because they fix carbon through photosynthesis, which is effectively stocked as peat^3^. Furthermore, *Sphagnum* mosses filter inorganic nutrients and help to maintain nutrient-poor environments, which is important for plant succession in peatlands^4–6^.

In recent years, substantial changes in *Sphagnum*-dominated peatlands have become serious owing to environmental changes, including global warming^7^ and increased atmospheric nitrogen (N) deposition^8^. Specifically, negative effects of temperature on *Sphagnum* mosses vulnerable to warming have been reported^9^. One meta-analysis indicated that warming promotes *Sphagnum* growth in general when water availability is not limited^10^; however, water availability can also be altered by global warming in that warming causes changes in precipitation patterns, including an increase in the mean length of dry spells^11^. Thus, when water supply is not sufficient, drought stress associated with increased temperature negatively affects *Sphagnum* mosses^12,13^. Although these mosses are somewhat resilient to prolonged drought, once damaged, their recovery is likely to be difficult^14^. Regarding increased N deposition, it improves *Sphagnum* growth by enhancing photosynthetic rate^13^. However, this positive effect becomes negative once N-critical load is exceeded, owing to the direct toxic effects of high N concentration^9^. The N-critical loads are expected to be 5–40 kg ha^−1^ yr^−1^ for northern *Sphagnum* peatland at middle–high latitudes^15^.

Warming and N treatment experiments targeting mesotrophic *Sphagnum* mosses at mid-latitudes may contribute to the understanding of *Sphagnum* responses to these environmental changes. Previous investigations involving concurrent warming and N treatments have focused on *Sphagnum* mosses (e.g., *Sphagnum balticum*, *Sphagnum cuspidatum*, *Sphagnum fuscum*, and *Sphagnum magellanicum*)^9,12,13^, which mainly dominate peatlands at above mid–high latitudes (46–68° N)^9^. These studies could not establish the additive positive effects of warming and N treatment on *Sphagnum* mosses. However, such additive effects may be observed in mesotrophic *Sphagnum* species with more southern distribution, which have adapted to warmer climates and increased N deposition. Among *Sphagnum* mosses in peatlands, *Sphagnum papillosum* Lindb. is a potential candidate for examining possible additive effects because this species primarily dominates at mid-latitudes (30–45° N)^9^ and exhibits higher N-use efficiency than ombrotrophic *Sphagnum* species^16^.

Changes in biomass production and the associated ecophysiological activities are useful for evaluating the effects of warming and N treatments on *S. papillosum*. The changes in biomass production are directly linked to the positive and negative effects of these treatments on *Sphagnum* growth^9,12^. The mechanisms underlying *Sphagnum* responses to warming and N treatment can be further assessed through photosynthetic performance and characteristics of carbon assimilation, such as a maximum quantum yield of photosystem II (Fv/Fm ratio) and a stable carbon isotopic ratio (δ^13^C). For example, higher N availability enhances the photosynthetic rate through an increase of chlorophyll content^13^. The enhanced photosynthetic rate is reflected in chlorophyll fluorescence parameters that represent plant health and vigor (e.g., Fv/Fm)^17^. Temperature increases the photosynthetic rate by activating enzymes related to chlorophyll production^18^; it further governs the carbon assimilation rate during photosynthesis when the water supply is sufficient^19^. The enhanced carbon assimilation rate is accompanied by ^13^C enrichment, resulting in increased δ^13^C levels^19^.

In this study, possible responses of *S. papillosum* to warming and N treatments were investigated using realistic treatment scenarios. Importantly, these scenarios included a decline in atmospheric N deposition, explained as follows. Contrary to its global increasing trend, atmospheric N deposition may remain stable or decrease in areas such as North America and East Asia by 2100^20^. Under this scenario, a decrease in N deposition may have a negative impact on the growth of oligomesotrophic *Sphagnum* mosses (*S. papillosum*). Therefore, considering the above scenario, we investigated the following hypotheses:

- Hypothesis 1: simultaneous warming and N treatment impose additive positive effects on the biomass production of *S. papillosum*, given that this *Sphagnum* moss adapts to warmer climates and oligomesotrophic environments.
- Hypothesis 2: warming and N treatment increase both the Fv/Fm ratio and δ^13^C level through enhancing photosynthetic activities.
- Hypothesis 3: decreased N supply negatively affects the biomass production of *S. papillosum,* which grows in a mesotrophic environment.

## Results

### Effects of warming and N treatment on *Sphagnum* biomass

**Figure 1:**
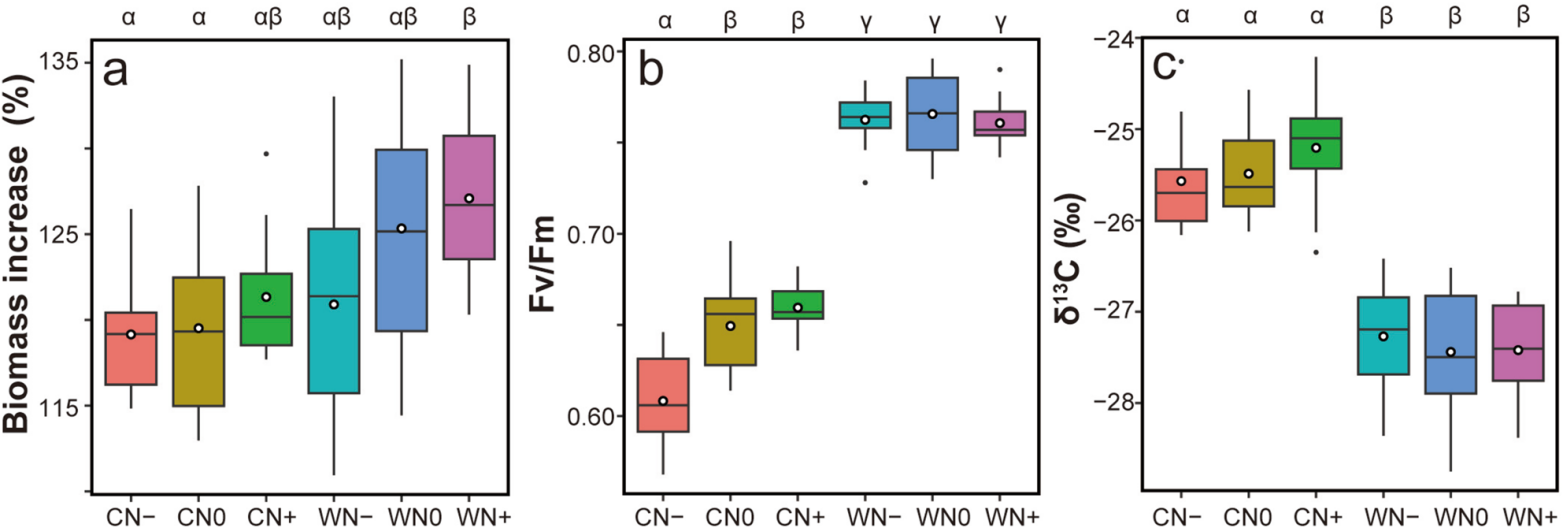
Effect of warming and N treatments on biomass production and ecophysiological traits. (**a**) Biomass increase (%), (**b**) Fv/Fm ratio, and (**c**) δ^13^C (‰) level. The upper and lower bounds of boxes represent the 75th and 25th percentiles, respectively. The middle bars and white dots represent the median and mean, respectively. The whiskers extend from the minimum to maximum values, or the minimum and maximum values within 1.5× the interquartile range. Statistically significant differences are indicated with different letters. Abbreviations: CN−, background temperature and low N level; CN0, background temperature and N level; CN+, background temperature and high N level; WN−, warmed temperature and low N level; WN0, warmed temperature and background N level; WN+, warmed temperature and high N level.

The average biomass increase was 119.2 ± 3.2%, 119.5 ± 4.8%, and 121.3 ± 3.6% (mean ± SD) in non-warmed and low N, control N, and high N treatment (CN−, CN0, CN+), respectively. Regarding the warmed plots, the biomass increase in low N, control N, and high N treatments (WN−, WN0, WN+) was 120.9 ± 6.4%, 125.3 ± 6.4%, and 127.1 ± 1.0%, respectively. Results of the two-way analysis of variance revealed that both warming and N treatments positively affected biomass production (Table 1). However, the interaction was not statistically significant. A post-hoc test showed that WN+ treatment resulted in a higher biomass increase than did CN0 and CN− treatment (Fig. 1a, Supplementary Table S1).

**Table 1:** Effects of warming and N treatment on *Sphagnum* biomass increase assessed using two-way ANOVA.

Factors	Df	Sum sq	Mean sq	*F* value	*p*
Warming	1	355.2	355.2	13.218	0.000544
Nitrogen	2	211.9	105.9	3.943	0.024119
Warming × Nitrogen	2	65.2	32.6	1.214	0.303494
Residuals	66	1773.3	26.9

### Chlorophyll fluorescence

The Fv/Fm ratio in the non-warmed and warmed plots was 0.64 ± 0.03 and 0.76 ± 0.02 (mean ± SD), respectively. Compared to non-warming treatment, warming treatment significantly enhanced Fv/Fm (Fig. 1b, Supplementary Table S1). In warmed plots, N treatment did not affect Fv/Fm, whereas in non-warmed plots, CN− treatment resulted in significantly lower Fv/Fm than did CN0 and CN+ (CN−: 0.608 ± 0.026, CN0: 0.649 ± 0.023, and CN+: 0.660 ± 0.013).

### Stable carbon isotope ratio

The value of δ^13^C in the non-warmed and warmed plots was − 25.4 ± 0.55‰ and − 27.4 ± 0.59‰ (mean ± SD), respectively. Warming treatment resulted in a significant decrease in δ^13^C compared to non-warming treatment (Fig. 1c, Supplementary Table S1). However, N treatment did not affect δ^13^C in either warmed or non-warmed plots.

## Discussion

We investigated the effects of simultaneous warming and N treatment on growth and ecophysiological activity of *S. papillosum*. The simultaneous warming and high N treatment resulted in enhanced biomass increase compared to the control, whereas a single treatment (warming or high N treatment) did not. These results validate Hypothesis 1, which predicted the additive effects of these treatments on biomass production. Focusing on the ecophysiological activities, warming treatment increased and decreased the Fv/Fm ratio and δ^13^C level, respectively; whereas N treatment only increased Fv/Fm in the non-warmed plots. These results do not support Hypothesis 2, because it predicted that both warming and N treatment would increase the Fv/Fm ratio and δ^13^C level. The low N treatment decreased Fv/Fm in non-warmed plots, validating Hypothesis 3, which predicted a negative influence of low N treatment on *S. papillosum*.

The increased biomass production due to warming treatment can be attributed to enhanced photosynthetic activity, as evidenced by the higher Fv/Fm ratio in the warmed samples than in the non-warmed ones. Warming may induce the activity of enzymes involved in chlorophyll production^18^. Given that the photosynthetic efficiency of *S. papillosum* increases with temperature up to 30–35 °C^21^, the warming treatment used in this study (19.6 °C) falls within the temperature range that enhances photosynthetic performance. Notably, the water status of *S. papillosum* may also be impacted by the warming treatment, as indicated by the significantly lower δ^13^C values observed than those seen with the control. In drier conditions, δ^13^C of *Sphagnum* tends to be more negative due to the enhanced ease of CO_2_ diffusion through photosynthetic cells; consequently, ^13^C discrimination during carbon assimilation is more pronounced^22^. These responses indicate that warming can reduce the moisture content in photosynthetic cells through increased water evaporation; however, this water stress is too low to reduce the photosynthetic rate. This enhanced water evaporation can facilitate CO_2_ diffusion within photosynthetic cells, resulting in the depletion of ^13^C in the assimilated carbon.

WN+ treatment did not have a significant positive effect on Fv/Fm in the warmed plots, although only WN+ significantly enhanced biomass production compared to the CN0 treatment. This result suggests that high N treatment may affect indices for photosynthetic performance other than Fv/Fm (e.g., maximum photosynthetic rate and chlorophyll content), resulting in additive effects of warming and N treatment on *S. papillosum*. This possibility is supported by the different responses of these indices to N treatment from that of Fv/Fm^13^. Furthermore, it may be possible that Fv/Fm did not respond only to N treatment because it reflects comprehensive environmental stresses^23^. The comparison of chlorophyll fluorescence revealed that the Fv/Fm ratio was significantly lower in CN− compared to CN0 treatment. This observation implies that a reduction in N deposition may have an adverse impact on the growth of *S. papillosum*. Given that changes in chlorophyll fluorescence serve as an early indicator of plant stress before irreversible damage^23^, a decrease in atmospheric N deposition can first cause a decrease in Fv/Fm, which may subsequently lead to a lower biomass production than that at current production. By contrast, Fv/Fm did not differ between the N treatments in the warmed plots. The enhanced photosynthetic activity due to warming may mask the different effects of N treatment on Fv/Fm between WN− and WN0.

Based on these results, the positive additive effects of warming and N treatment on biomass increase were confirmed in this study. Previous warming and N treatment studies have not revealed such an additive effect^6,9,12,13^. This finding can be attributed to both the characteristics of the *Sphagnum* moss used in this study and the amount of N supply. As hypothesized, both warming and N treatment may be beneficial for *S. papillosum,* which has adapted to warmer climates and mesotrophic environments. Furthermore, the amount of N addition in this study (5.0–14.9 kg N ha^−1^ yr^−1^) was adjusted to lower levels than in previous studies (28–40 kg N ha^−1^ yr^−1^)^6,9,12,13^ due to the low background level of N deposition (9.9 kg N ha^−1^ yr^−1^) at the study site. Hence, even the high N treatment in this experiment did not reach N levels toxic to *S. papillosum*.

We predicted the changes in *S. papillosum* biomass production at the study site based on the obtained results. According to the possible future scenario around the study area (+ 4.8 °C under the SSP5-8.5 scenario^24^ and 0.56 N deposition decrease^20^), WN− treatment can simulate the future environment while CN0 is regarded as current environment. The difference of *Sphagnum* biomass production between WN− and CN0 is only + 1.4%. This finding implies a small increase in *S. papillosum* biomass under the possible future scenario at the study site. Applying these results to other regions, biomass increase can be more pronounced (+ 7.6%) in areas with N deposition equivariant to 1.5 times higher than that of the study site (14.9 kg N ha^−1^ yr^−1^). However, in an actual ecosystem, the biomass increase may be offset by associated environmental changes, such as increased evaporation^12^, decline of the water table^25^, and the overgrowth of vascular plants^6,26^.

## Conclusion

Simultaneous warming and N addition were shown to have additively positive effects on *S. papillosum*. This additive effect may be attributed to the ecological characteristics of *S. papillosum* and the experimental design of the low N treatment. These results contribute to our understanding of the responses of *Sphagnum* mosses to warming and N treatments. *Sphagnum* production at the study area can be expected to slightly increase under possible future climate scenarios, according to the results of this study. However, the actual responses of *S. papillosum* may be more complicated than those obtained from laboratory experiments. A meta-analysis that includes these factors will be useful for predicting changes in *Sphagnum*-dominated peatlands.

## Methods

### Experimental design

*S. papillosum* samples were collected from a *Sphagnum*-dominated peatland in Nagano Prefecture, Japan (36.7832° N, 137.8140° E) with permission from the appropriate governing bodies^27,28^. *Sphagnum* samples were collected from monospecific communities. The identification of *Sphagnum* was conducted by the author. Voucher specimens were deposited in an herbarium of Fukui Prefectural University. The experiments were conducted in growth chambers (LH-60FL12-DT; Nippon Medical & Chemical Instruments Co., Ltd., Osaka, Japan) for over 3 months, which is equivalent to the growth period at the study site. *Sphagnum* mosses were cultivated under 12-h light (8000 lx) and 12-h dark conditions. *Sphagnum* samples were cut to a length of 5 cm and placed in a pot (diameter, 6 cm; height, 5.5 cm) set in a plastic container. The water level in each container was maintained at a height of 1 cm, such that the mosses did not experience drought. This experimental design was conducted considering that annual precipitation may not significantly change in Japan by 2100, yet changes in precipitation patterns are difficult to predict^29^. The position of the pots was randomly changed weekly. *Sphagnum* pots (72) were divided into six treatments, each with 12 replicate blocks. Two temperature treatments (warmed, W; non-warmed, C) and three levels of N supply (low N; N−, control N; N0, high N; N+) were adopted. The temperature treatments included control (C: 14.8 °C) and warming (W: 19.6 °C). The control temperature was determined based on the average temperature near the study site (Happo-one) during the growing season over 10 years^30^, whereas the warming temperature was simulated using a possible warming scenario (+ 4.8 °C) relative to 1995–2014 under the SSP5-8.5 scenario in the Intergovernmental Panel on Climate Change sixth assessment report^24^.

### Nitrogen and other nutrients

The N supply doses were based on the annual N deposition near the study site (9.9 kg N ha^−1^ yr^−1^)^31^ and estimated changes in atmospheric N deposition in East Asia by 2100 (0.56–1.35 times as much as in 2000)^20^. For ease of comparison, the levels of N addition were set at 0.5 and 1.5 times of the background N level in this experiment. The N control treatment (N0) was one-fourth of the N deposition (2.48 kg N ha^−1^ equivalent to three months of annual N deposition), and 1.5 and 0.5 times of the N0 amount were applied to represent increased (N+: 3.71 kg N ha^−1^) and decreased (N−: 1.24 kg N ha^−1^) levels. Nitrogen (NH_4_NO_3_) and other nutrients were dissolved in distilled water and supplied to the *Sphagnum* samples via spray. The types and concentrations of the additional elements (P, K, S, Ca, Mg, Na, Fe, Mn, B, Zn, Mo, Cu, Cl, and Ni) dissolved in the water used for irrigation were determined based on their average values in precipitation collected near the study site (P, K, S, Mg, Ca, and Na)^30^ or over Japan (other nutrients)^32–36^ in cases where no measurement of element concentration was reported near the study site. The composition of the synthetic precipitation was adjusted using KH_2_PO_4_, KCl, CaSO_4_·2H_2_O, NaSO_4_, MgCl_2_·6H_2_O, FeCl_3_·6H_2_O, MnCl_2_·4H_2_O, H_2_BO_3_, ZnCl_2_, Na_2_MoO_4_·2H_2_O, CuCl_2_·2H_2_O, NaCl, and NiCl_2_·6H_2_O. The quantity and frequency of watering were determined based on the total precipitation and number of rainy days during the growing season at the study site. The average precipitation and rainy days during the growing season in 10 years were 887 mm/m^2^ and 40.6 days, respectively^30^. Therefore, the amount of water dissolving N and other nutrients totaling 887 mm/m^2^ was added to *Sphagnum* samples on alternate days (45 times) during the three-month experimental duration (Supplementary Fig. S1).

### Biomass, chlorophyll fluorescence, and stable carbon isotope ratio

Changes in biomass production (biomass increase) were calculated as the weight gain relative to the initial weight (%). The weight of the fully wet samples was measured at the start and end of the experiment because the drying of samples can affect physiological activity. Before weight measurement, *Sphagnum* samples were fully soaked in water for 30 s and then left at room temperature for 24 h to allow removal of excess water. A preliminary experiment confirmed a significant correlation between wet and dry weights and a simple linear model was best-fitted for this correlation.

FluorPen FP110 (Photon Systems Instruments, Drásov, Czech Republic) was used to measure the Fv/Fm ratio. Preliminary experiments were used to calibrate the optimum flash pulse, super pulse, and actinic pulse intensities at 20%, 30%, and 300 uE, respectively. The dark adaptation time was adjusted to 30 min. The Fv/Fm ratio was measured for all *Sphagnum* pots before harvest.

*Sphagnum* samples were oven-dried at 40 ℃ for more than 48 h until a constant weight was obtained. The samples were homogenized using a Retsch mill (Retsch MM 400, Retsch GmbH, Germany). Approximately 3 mg of each homogenized sample was used to measure the ^13^C/^12^C ratio by using an elemental analyzer equipped with an isotope ratio mass spectrometer (Flash EA 1112-Conflo IV-Delta V Advantage; Thermo Fisher Scientific, Waltham, MA, USA) at the Research Institute for Humanity and Nature (Kyoto, Japan). The following equation was used to calculate δ^13^C: [(Rsample − Rstandard)/Rstandard] × 1000, where R = ^13^C/^12^C. The obtained δ^13^C values were corrected using standard reference materials (CERKU06 and CERUK07)^37^.

### Statistics

The effects of warming and N treatment on changes in *Sphagnum* biomass were assessed using a two-way ANOVA with the main factors (warming: two levels, N treatment: three levels) and a significance level *p* < 0.05. A post-hoc Tukey–Kramer test was performed to compare biomass production among treatments. Before these analyses, normality and homoscedasticity were assessed. The comparison of Fv/Fm and δ^13^C values among treatments was performed using the Wilcoxon rank sum test using the Bonferroni correction because of the non-normality of the dataset for each treatment. All analyses were conducted using R version 4.3.0 (R base and “exactRankTests” packages)^38^.

### Ethical approval

Permission to collect *Sphagnum* samples was obtained from the appropriate governing bodies (Ministry of the Environment and Forestry Agency).

### Supplementary Information

Supplementary Figure S1.

Supplementary Table S1.

## Data Availability

All data used in this study are available in the supplementary materials.
