# Supplementary material for: Additive positive effect of warming and elevated nitrogen deposition on Sphagnum biomass production at mid-latitudes
Source: Sci Rep. 2024 Jul 22;14:16793. doi: 10.1038/s41598-024-67614-5 (PMC11263368; doi:10.1038/s41598-024-67614-5)
Supplement: Supplementary file 1 — Supplementary Figure S1. [file 41598_2024_67614_MOESM1_ESM.docx]

**
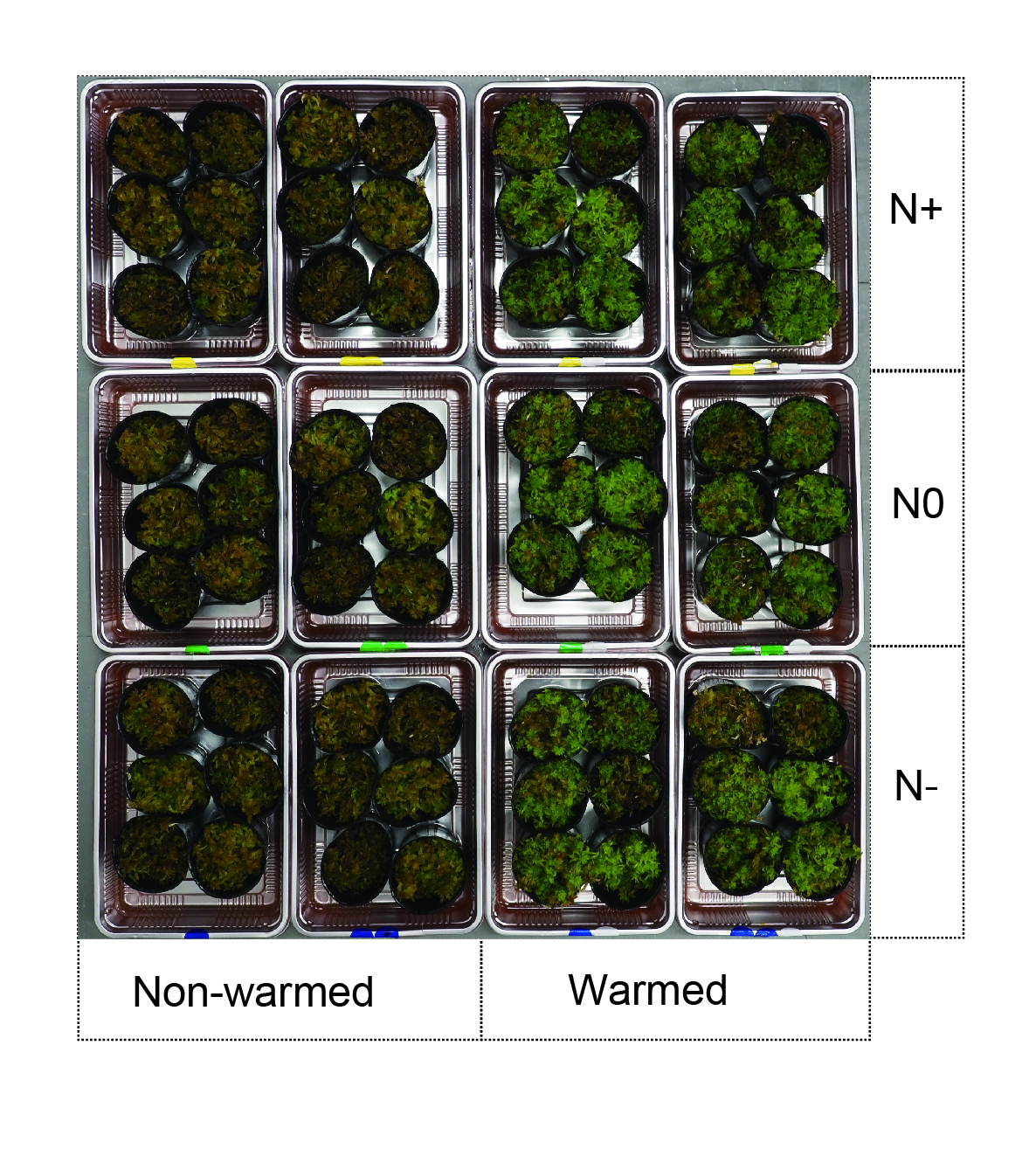
Supplementary Figure S1. *Sphagnum* pots after warming and N treatment for 3 months.** Abbreviations: CN−, background temperature and low N level; CN0, background temperature and N level; CN+, background temperature and high N level; WN−, warmed temperature and low N level; WN0, warmed temperature and background N level; WN+, warmed temperature and high N level.
